# Expression of the SARS-CoV-2 receptorACE2 in human heart is associated with uncontrolled diabetes, obesity, and activation of the renin angiotensin system

**DOI:** 10.1186/s12933-021-01275-w

**Published:** 2021-04-27

**Authors:** Michal Herman-Edelstein, Tali Guetta, Amir Barnea, Maayan Waldman, Naomi Ben-Dor, Yaron Barak, Ran Kornowski, Michael Arad, Edith Hochhauser, Dan Aravot

**Affiliations:** 1 Cardiac Research Laboratory, Felsenstein Medical Research Center, Sackler School of Medicine Tel-Aviv University, Tel Aviv, Israel; 2 Department of Cardiothoracic Surgery, Rabin Medical Center, Petach Tikva, Israel; 3 Department of Cardiology, Rabin Medical Center, 49100 Petach Tikva, Israel; 4 Leviev Heart Center, Sheba Medical Center, Tel Hashomer, Tel Aviv University, Tel Aviv, Israel; 5 Nephrology Department, Rabin Medical Center, Petach Tikva, Israel; 6 Sackler Faculty of Medicine, Tel Aviv University, Tel Aviv, Israel

**Keywords:** Corona virus 2 (SARS-CoV-2), Angiotensin-converting enzyme 2 receptor (ACE2), Heart, Diabetes, Renin–angiotensin system blockers

## Abstract

**Background:**

Diabetic and obese patients are at higher risk of severe disease and cardiac injury in corona virus 2 (SARS-CoV-2) infections. Cellular entry of SARS-CoV-2 is mainly via the angiotensin-converting enzyme 2 (ACE2) receptor, which is highly expressed in normal hearts. There is a disagreement regarding the effect of factors such as obesity and diabetes on ACE2 expression in the human heart and whether treatment with renin–angiotensin system inhibitors or anti-diabetic medications increases ACE2 expression and subsequently the susceptibility to infection. We designed this study to elucidate factors that control ACE2 expression in human serum, human heart biopsies, and mice.

**Methods:**

Right atrial appendage biopsies were collected from 79 patients that underwent coronary artery bypass graft (CABG) surgery. We investigated the alteration in *ACE2* mRNA and protein expression in heart tissue and serum. *ACE2* expression was compared with clinical risk factors: diabetes, obesity and different anti-hypertensive or anti-diabetic therapies. WT or db/db mice were infused with Angiotensin II (ATII), treated with different anti-diabetic drugs (Metformin, GLP1A and SGLT2i) were also tested.

**Results:**

*ACE2* gene expression was increased in diabetic hearts compared to non-diabetic hearts and was positively correlated with glycosylated hemoglobin (HbA1c), body mass index (BMI), and activation of the renin angiotensin system (RAS), and negatively correlated with ejection fraction. *ACE2* was not differentially expressed in patients who were on angiotensin converting enzyme inhibitors (ACEi) or angiotensin receptor blockers (ARBs) prior to the operation. We found no correlation between plasma free *ACE2* and cardiac tissue *ACE2* expression. Transmembrane serine protease 2 (*TMPRSS2),* metalloprotease *ADAM10* and *ADAM17* that facilitate viral-*ACE2* complex entry and degradation were increased in diabetic hearts. *ACE2* expression in mice was increased with ATII infusion and attenuated following anti-diabetic drugs treatment.

**Conclusion:**

Patients with uncontrolled diabetes or obesity with RAS activation have higher ACE2 expressions therefore are at higher risk for severe infection. Since ACEi or ARBs show no effect on *ACE2* expression in the heart further support their safety.

## Background

ACE2, the receptor via which SARS-CoV-2 enters the cells, is a known regulator of the renin angiotensin system (RAS) [1]. *ACE2* down regulates RAS activation by increasing ATII degradation into an anti-fibrotic: angiotensin (1–7) and by activating *MAS1* receptor axis [2]. Cells expressing *ACE2* are potential targets of SARS-CoV-2 infection*. ACE2* is a membrane‐bound enzyme that also circulate in the plasma as a consequence of proteolytic shedding events [3]. Enhanced *ACE2* shedding, resulting from RAS over-activation, is seen in the serum of diabetic patients and patients with failing heart [4–6], possibly making them more vulnerable to COVID19 infection [7]. However, as far as we know there is no data on the correlation between *ACE2* myocardial tissue expression to the concentrations of soluble ACE2 receptors in serum or plasma.

*ACE2* receptor is widely expressed in different tissues: lungs, kidneys, testis, skin and gastrointestinal tract and is highly expressed in the normal heart. The expression of *ACE2* in the heart is confined to the endothelium of the coronary arteries, cardiac myocytes, fibroblasts, epicardial adipocytes, and smooth muscle cells of the myocardial vessels, making the heart a major target to the coronavirus infection and injury [8–10].

Cardiac injuries from SARS-CoV-2 manifested by myocarditis and late fibrosis are commonly seen during severe COVID19 infection secondary to viral cell invasion and active viral replication in the cardiomyocyte [11, 12]. Certain patients are more vulnerable to SARS-CoV2 infection than others. Some will become extremely sick with inflammatory “cytokines storm” and cardiac injury, while others will express only mild symptoms or are asymptomatic. The increased risk factors include old age, male gender, cardiovascular disease, diabetes, and obesity [6, 13–15]. These risk factors are possibly secondary to dysregulation of the RAS and increased tissue *ACE2* expression.

Increased expression of *ACE2* is seen in tissues of diabetic and obese patients is likely to promote higher entry of SARS-CoV-2 into cells, thus increasing the susceptibility for infection and contributing to worse outcomes in these patients [7, 16].

Angiotensin-converting enzyme inhibitors (ACEi) and angiotensin II receptor blockers (ARBs), which are widely used in diabetes and heart failure, increase the levels of ATII and could indirectly activate ACE2 [17, 18]. Since the beginning of COVID-19 pandemic, there was a concern regarding the effect of ACEi or ARBs that elevate *ACE2* expression that may increase the susceptibility for infection in patients receiving these medications [8]. However recent data showed that patients continuing ACEi or ARB have better prognosis with less inflammation during COVID-19 infection compared with patients that discontinues RAS blockade [19, 20]. Furthermore, there is disagreement on the effect of diabetes and level glycemic control or uses of different anti-diabetic medications on the expression of ACE2 [21].

The aim of our study is to elucidate the factors that control the cardiac *ACE2* expression in diabetic patients and db/db mice treated with ATII infusion and different anti diabetic drugs.

## Methods

### Subjects and clinic evaluation

Diabetic patients (n = 57) and non-diabetic (n = 22) undergoing isolated CABG operation (between 2015 and 2020) at the Department of Cardiothoracic Surgery, Rabin Medical Center (RMC), were included in this study. The study was approved by the Institutional Ethics Committee according to the declaration of Helsinki (#0017-15-RMC), all the patients sign an informed consent.

Seventy-nine atrial tissues samples obtained during the cannulation of the right auricle, were cryopreserved at – 80 °C for mRNA or protein extraction and in 4% formaldehyde for later processing to paraffin blocks for IHC staining. Serum was collected from all the patients before the CABG operation.

Patient’s medical records were reviewed and clinical parameters including the demographics, co-morbidities, echocardiographic parameters, laboratory parameters and medications, with special attention to ACEi, ARB’s and various anti-diabetic drugs were reported.

### Mouse model

The animal experiments were approved by the institutional animal care and use committee of Tel Aviv University (01-18-001).

Male mice with homozygous leptin receptor deficiency (db/db) that develops obesity and T2DM were studied. These mice develop cardiomyopathy, when exposed to ATII stress [23]. The genetic status was confirmed by PCR as described [23]. Mice were maintained in a pathogen free facility on regular rodent chow with free access to water and 12-hlight and dark cycles. For angiotensin II (ATII) infusion mice wild type (WT) or db/db mice (n = 6 − 8/group), 12–14 weeks old, were anesthetized with 2% isoflurane and an ALZET osmotic pump (Durect Corp., Cupertino, CA, USA) was subcutaneously implanted into each mouse. The osmotic pumps infused ATII (Sigma-Aldrich, St. Louis, MO, USA) at a rate of 1000 ng·kg·min. After competing 4 weeks infusion mice underwent echocardiography and were sacrificed. Blood was drawn from the inferior vena cava for biochemistry; the heart was rapidly removed and processed for histology, RNA extraction and protein studies [22].

Drugs were administered concomitantly with ATII. Dapagliflozin (DAPA, ASTRAZENECA, DE 19850-5437 USA) and metformin (Sigma Aldrich) were administered in drinking water at 1.5 mg/kg/day [23] and 300 mg/kg/day [24], respectively. Dulaglutide (Trulicity, Eli Lilly, USA, was injected SC 0.6 mg/kg twice weekly [25].

The following groups were studied:

1. WT, saline infusion for 1 month.
2. WT, AngII infusion for 1 month.
3. db/db, saline infusion for 1 month.
4. db/db, AngII infusion for 1 month.
5. db/db, saline infusion + DAPA for 1 month.
6. db/db, AngII infusion + Metformin for 1 month.
7. db/db, AngII I infusion + Dulaglutide for 1 month.

### RNA extraction and quantitative real-time PCR

Total RNA was purified from frozen human and murine heart samples using TRIzol (Ambion, Austin, TX, USA) according to the manufacturer’s instructions. cDNA was synthesized from total RNA using qScript cDNA Synthesis Kit (Quantabio, USA) according to the manufacturer’s protocol. QRT-PCR, was performed as described previously [23], using the TaqMan or Syber system and analyzed by StepOnePlus™ Real-Time PCR Systems (Applied Biosystems, CA, USA). Gene expression was normalized to endogenous control genes: 18S, *RPLPO* (human study) and B2M (mouse study). Results are presented normalized to *RPLPO or B2M*, the most consistent expression. Relative mRNA level of the different genes was normalized to the level of endogenous control gene. The following primers were used:

Primers sequences:

Human:

ACE2: 5′-GCTTATCCTCACTTTGATGCTTTG-3′.

5′-GCCACTGCTCAACTACTTTG-3′.

ACE1: 5′-CACCAATGACACGGAAAGTG-3′.

5′-AGGAGGACAGATCCCCTGAT-3′.

TMPRSS2: 5′-CTCTCCCTAACCCCTTGTCC-3′.

5′-AGAGGTGACAGCTCCATGCT-3′.

FURIN: 5′-ACAACTATGGGACGCTGACC-3′.

5′ TGGACACAGCTCTTCTGGTG-3′.

ADAM17: 5′ TTGGGTCTGTCCTGGTTTTC-3′.

5′-CGCAGGAAAGGGTTTGATAA-3′.

ADAMS10: 5′-CAGTTAGCGTCTCATGTGTCC-3′.

5′ GTAGTAATCCAAAGTTGCCTCCT-3′.

MAS1: 5′-CAGGGAAATGTGGTGTAGGTT-3′.

5′-CCATCATTATATTCCTCATCTTCGC-3′.

AGTR1: 5′-CCAGTTTCCAAAGGGCAGTA.

3′ 5′-CCATCTTACGGGCATTGTTT-3′.

RPLP0: 5′-TGTCTGCTCCCACAATGAAAC-3′.

5′ TCGTCTTTAAACCCTGCGTG-3′.

Mouse: mACE2: 5′-GGCTTATCCTCACTTTAATGCTTT-3′.

5′-TGCTCAATTACTTCCAACCGT-3.

mACE1-5′-TTCACAGAGGTACACTGCTTG-3′.

5′-ACAAGTCGATGTTAGAGAAGCC-3′.

mTMPRSS2-5′-GCTAAACACAGCGATTTCTTAGAC-3′.

5′-GATTACAACGCAAGCCTCAAC 3′

mFurin: 5′-CATCAGTCACCTCGCCATC-3′.

5′-CGGTACACACAGATGAATGACA-3′.

mADAMS17: 5′-GATGTCGTAGTCTGAGAGCAA-3′.

5′-CTTTGGTGCCTTTCGTCCT-3′.

mADAMS10: 5′-TGATGCCTGTGTTCAATGACT-3′.

5′-TGATGGTGTTCTTGGTCTGG-3′.

B2M-0.5′-GGG TGG AAC TGT GTT ACG TAG-3′.

5′-TGG TCT TTC TGG TGC TTG TC-3′.

### ACE2 protein in serum and tissue

We tested *ACE2* protein levels in 32 cardiac tissue biopsies and serum using sandwich *ACE2* ELISA kit from BioVision, USA (catalog# E4528-100). According to the manufacturer’s protocol in 1 mg tissue protein or 1 ml serum and compared free serum ACE2 levels to tissue levels.

### ACE1 activity

We tested *ACE1* protein activity levels in 32 serum using Angiotensin I Converting Enzyme Activity (ACE1) Assay Kit (Fluorometric) from BioVision USA (catalog# K227-100).

### Immunohistochemistry (IHC) staining

IHC was performed on 4 μm FFPE human heart biopsies using anti-ACE2 antibody (cat. no. ab15348; Abcam, Cambridge, MA, USA; dilution, 1:200) and the HRP one-step polymer detection system: anti-mouse-rabbit-rat (ZyoChem Plus) and DAB. ACE2, Biopsies (n = 40) were analyzed and captured together under the same setting. ACE2 quantification was made according to user guidelines of imageJ. Tissue area was marked and the red channel threshold for all the biopsies was defined. The ACE2 + signal was calculated only within the cardiac tissue and normalized to the total area.

Masson’s trichrome stain kit (Bio-optica Milano, Italy) was used for fibrosis staining. We randomly selected 10 fields of heart sections at × 20 magnification by light microscopy and analyzed % era by ImageJ.

### Statistical analysis

ANOVA and two-tailed Student’s t-test for independent data was performed. Linear correlations were calculated using GraphPad software. Correlation graph include 95% confidence interval line. *P* < 0.05 was considered significant.

## Results

### Up-regulation of the ACE2 in human diabetic heart

**Fig. 1:**
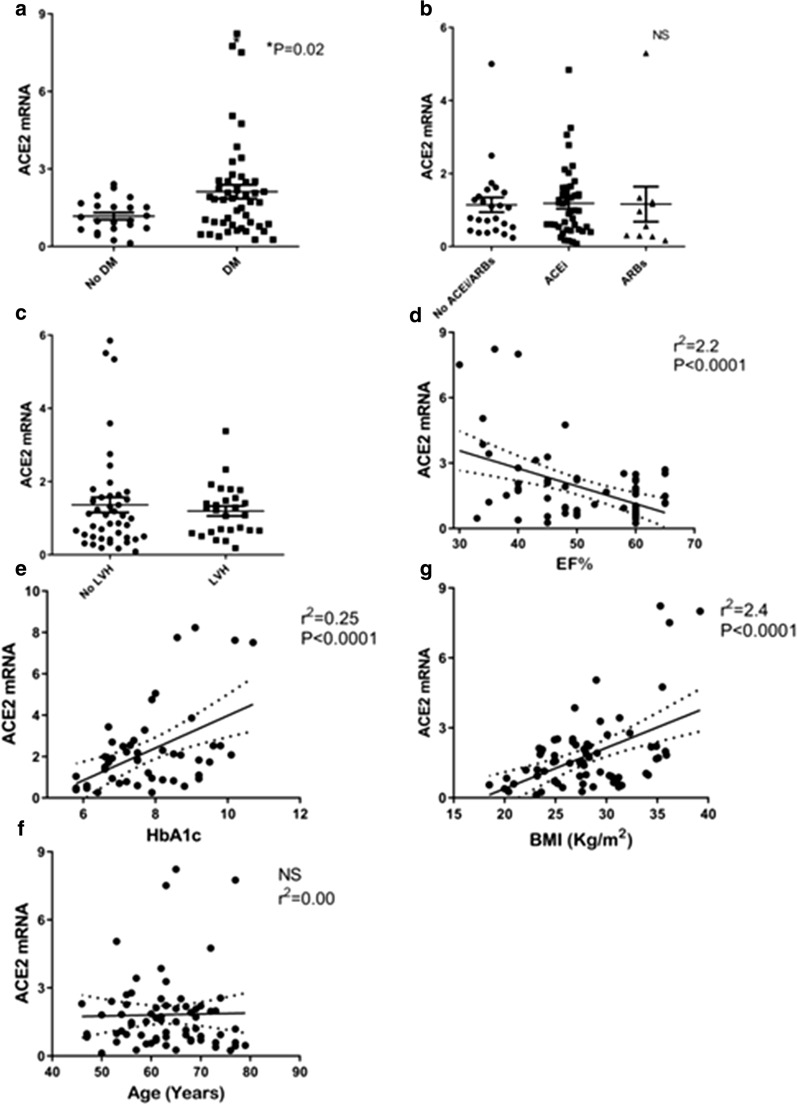
ACE2 mRNA is highly expressed in cardiac atrial biopsies and increased in cardiac biopsies of diabetic patients. **a** ACE2 mRNA expression is increased in human diabetic cardiac biopsies *vs* non-diabetic patients. *p = 0.02. **b** ACE2 mRNA expression is not significantly different between human cardiac biopsies of patients on ACEi, ARBs or no RAS blockers prior to the CABG operation. **c** ACE2 mRNA expression is not significantly different between hypertrophic heart vs no hypertrophic heart. **d** ACE2 mRNA expression in the human cardiac biopsies is positively correlated with HbA1c. **e** ACE2 mRNA expression in human cardiac biopsies is inversely correlated with fractional shortening (FS). **f** ACE2 mRNA expression in the human cardiac biopsies is positively correlated with BMI measured kg/m^2^ as measured prior to the operation. **g** There was no correlation between patients' age and ACE2 mRNA expression in the cardiac biopsies. RPLPO gene was used as the internal control. Values are shown as mean ± SEM. P value evaluated with paired Student’s t-test, *p < 0.05

**Fig. 2:**
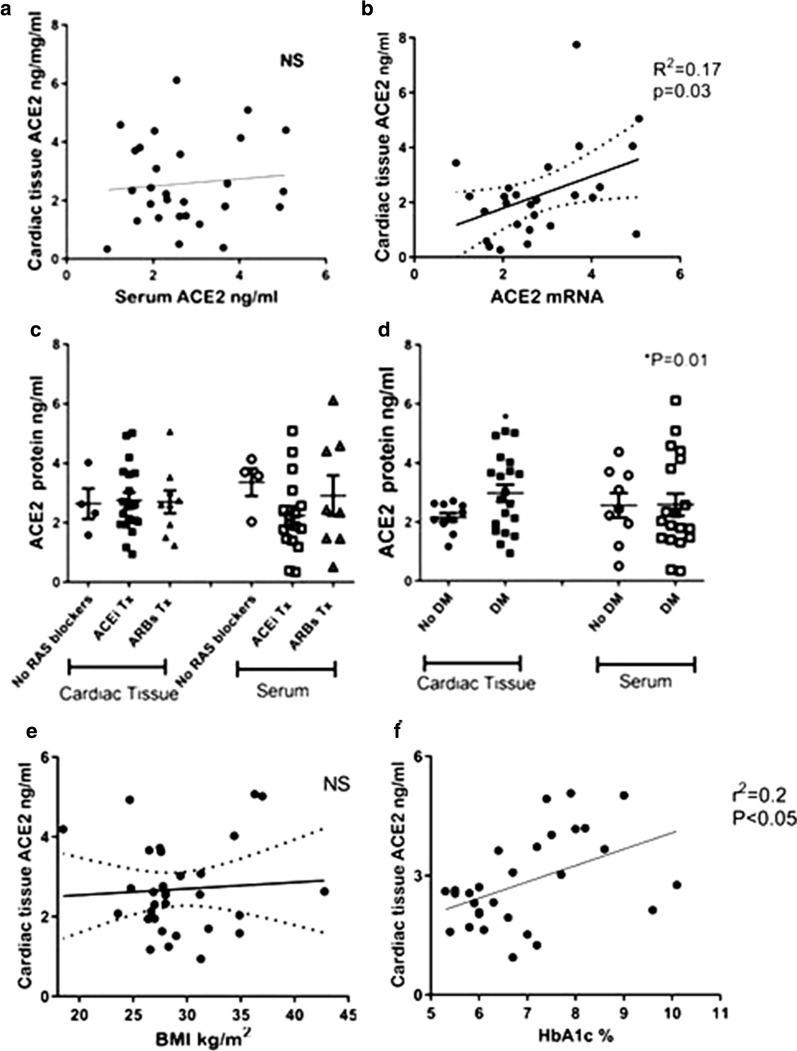
ACE2 protein expression in cardiac biopsies and serum. **a** There was no correlation between cardiac tissue protein levels measured by Elisa kit (ng per mg protein sample of cardiac biopsies) to ACE2 in 1 ml/serum sample from the same patient collected before the operation. **b** *ACE2* mRNA expression in the human cardiac biopsies was positively correlated with protein levels (n = 32). **c** *ACE2* protein in cardiac tissue or in patient’s serum levels were not significantly different between human cardiac biopsies of patients on ACEi, ARBs or no RAS blockers prior to the CABG operation. **d** *ACE2* protein levels were increased in human diabetic cardiac biopsies *vs* non-diabetic patients. *p = 0.01. Serum *ACE2* protein levels were not significantly different between diabetic *vs* non-diabetic. **e** *ACE2* protein levels in cardiac tissue from human cardiac biopsies positively correlated with HbA1c. **f** *ACE2* protein levels in cardiac tissue from human cardiac biopsies positively correlated with BMI

We first studied the relationship between diabetes-related clinical variables and ACE2 expression in heart tissues. We compared *ACE2* expression levels between diabetic *vs* non-diabetic patients (Table 1). We found that *ACE2* was up-regulated in diabetic hearts compared with non-diabetic ischemic hearts. ACE2 was assessed by mRNA as well as protein expression (Figs. 1, 2).

**Table 1:** Patients clinical characteristics

			Non diabetics (N = 22)	Diabetics (N = 57)
Gender (male N %)			21 (95%)	49 (86%)
Age (years)			62.7 ± 8.2	63.4 ± 8.4
BMI (kg/m^2^)			27.8 ± 5.1	28.5 ± 4.3
HbA1C (%)			5.6 ± 0.3	7.8 ± 1.4**
Glucose( mg/dL)			101.9 ± 27.7	168.1 ± 54.1**
EF (%)				
Normal			11 (50%)	22 (39%)
Mild			8(36%)	26 (46%)
Moderate			3 (14%)	7 (20%)
Severe			0	1 (2%)
LV Hypertrophy (N%)			7 (31%)	24 (42%)
S/P MI			13 (54.1%)	41 (54.2%)
Total Cholesterol (mg/dL)			136.3 ± 49.1	140.9 ± 41.7
HDL (mg/dL)			37 ± 7.9	37.8 ± 11.7
Triglycerides (mg/dL)			107.4 ± 33	169.8 ± 122*
LDL cholesterol (mg/dL)			76.5 ± 39.0	70.7 ± 31.0
Creatinine (mg/dL)			0.9 ± 0.3	0.39 ± 0.3
Hypertension (N %)			18 (75%)	48 (85%)
RAS blockers (N %)				
ACEi			15(68%)	25 (44%)
ARBs			3 (13%)	14 (25%)
Spironolacton			1 (4.5%)	2(3%)
Non			3(13%)	15 (26%)
Anti-diabetic Tx (N %)				
Insulin			0	21 (37%)
Met			0	39 (68%)
SGLT2i			0	9 (16%)
GLP1			0	5 (9%)
DPP4			0	5 (9%)
Sulfanilurea			0	13 (22%)

Values are shown as mean ± SD

*p < 0.05, **p < 0.005

*EF* ejection fraction, *s/p MI* state post-myocardial infarction, *LV* left ventricular, *Tx* treatment

The role of glycemic control was studied with correlation between HbA1c and *ACE2* mRNA and protein expression. A significant correlation was observed between HbA1c and *ACE2* mRNA (r^2^ = 0.24, P < 0.005) (Fig. 1d) and HbA1c and *ACE2* protein (r^2^ = 0.14, P = 0.04) (Fig. 2e) suggesting that glycemic control may affect ACE2 expression in the heart.

**Fig. 3:**
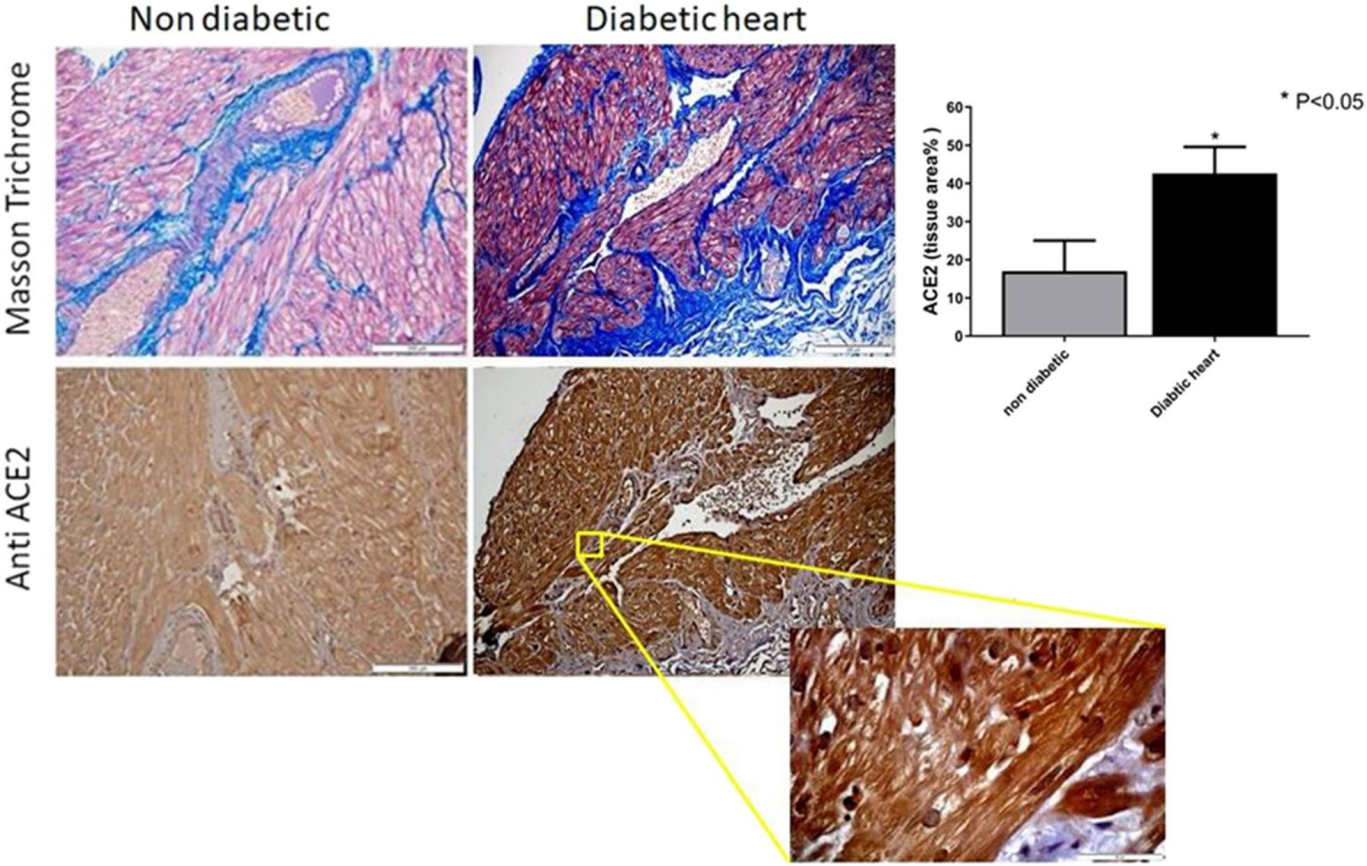
*ACE2* immunofluorescence microscopy indicates the increased expression in diabetic cardiomyopathy. Masson Trichrome staining for fibrotic tissue (blue) (× 20) (upper panel)*, ACE2* immunofluorescence microscopy (lower panel) and quantification of IHC staining (% tissue area). ACE2 staining is also shown also in higher magnification (× 100)

IHC staining showed that *ACE2* protein was highly expressed in the myocardium and endothelial cells. Higher *ACE2* was observed in the diabetic heart tissues compared with non-diabetic (Fig. 3). We then assessed the correlation between the left ventricular ejection fraction (EF) and ACE2. We demonstrate a negative correlation between *ACE2* mRNA expression and EF (Fig. 1e). On the other hand we found no significant correlation between *ACE2* mRNA and the presence of left ventricular hypertrophy (LVH) as assessed by echocardiography (Fig. 1c).

### Effect of obesity on ACE2 expression

Obesity is a well characterized risk factor for COVID19 infection and outcome. We studied the relationship between the BMI and ACE2 expression (Fig. 1e). There was a significant correlation between BMI and *ACE2* mRNA expression, but not with ACE2 protein expression (Figs. 1f, 2f, respectively).

### Effect of RAS blockers, on ACE2 expression

To study the effect of RAS blockade we divided the patients according to therapy by ACEi, ARBs or none prior to the operation.

Seventy percent of both groups were on RAS blockade (Table 1). No correlation was found between *ACE2* gene expression and the use of RAS blockers, suggesting that ARBs/ACE inhibitors treatment alone do not affect *ACE2* expression (Figs. 1b, 2b).

### Serum free ACE2 and cardiac tissue levels

It was suggested that serum ACE2 may be a biomarker of COVID-19 disease severity and represent tissue expression [26–28]. We found no correlation between serum free *ACE2* and cardiac tissue levels (Fig. 2a).

### Expression of genes effecting ACE2 trafficking

**Fig. 4:**
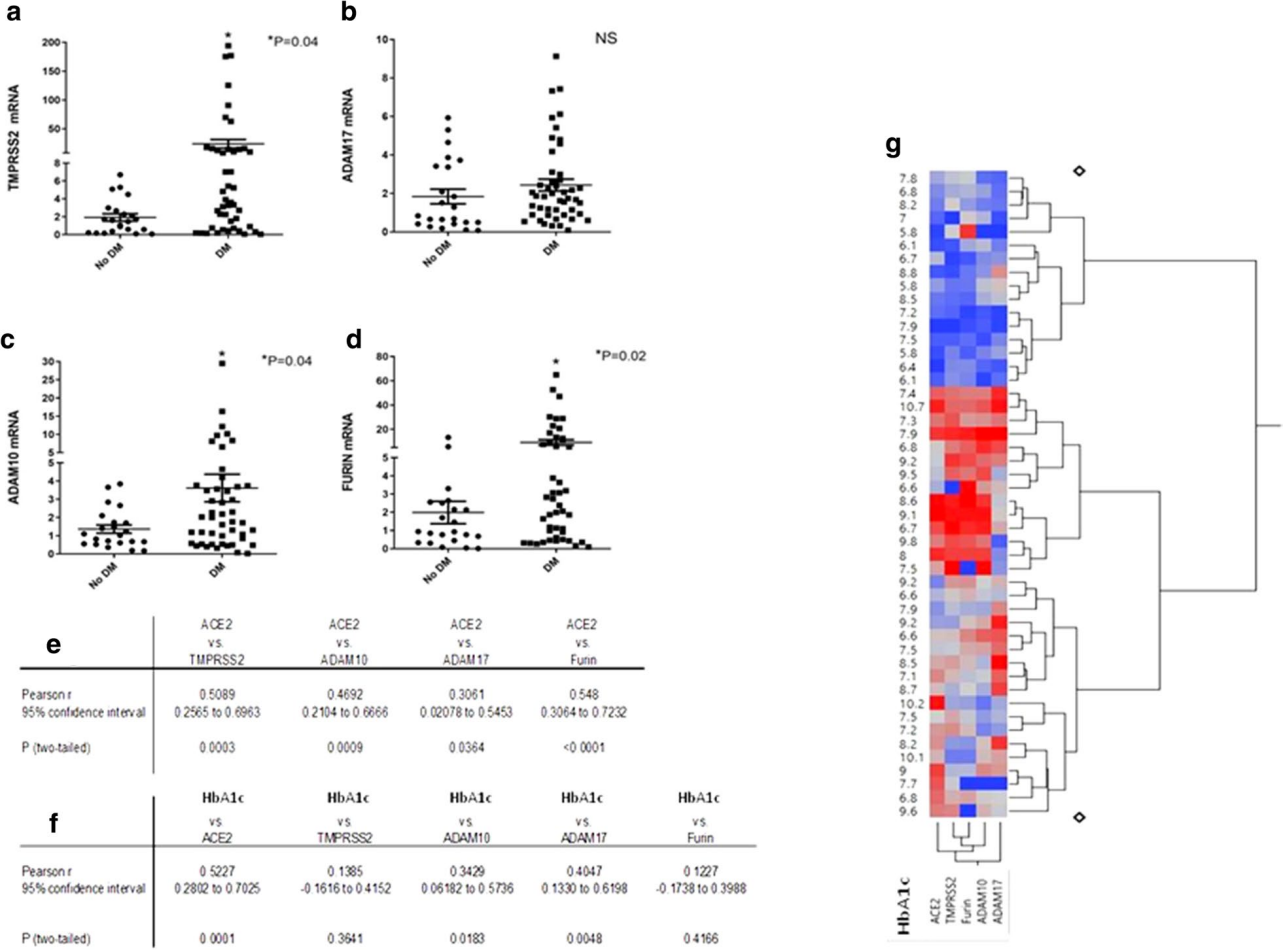
Expressions of genes affecting ACE2 internalization are increased in diabetic cardiac biopsies. **a** *TMPRSS2* mRNA. **b** *ADAM17* mRNA. **c** *ADAM10* mRNA. **d** *Furin* mRNA. Values are shown as mean ± SEM P value evaluated with paired Student's t-test, *p < 0.05. **e**–**g** Table + heat map correlation between ACE2 and proteases and HbA1c and ACE2 and the trafficking proteases

Because there was only a weak correlation between ACE2 tissue mRNA and its protein level (r^2^ = 0.17, p = 0.03) (Fig. 2b) we examined the expression of different genes affecting *ACE2* receptor cellular entry and trafficking: *ADAM17, ADAM10, TMPRSS2* and *Furin.* SARS-CoV-2 ACE2 entry to the cells requires proteolitic activity of Transmembrane serine protease 2 (*TPMRSS*2) that is expressed on ACE2-expressing cells in the heart, facilitating cleavage of the spike protein. *ADAM17* and also *ADAM10* cause proteolytic shedding of *ACE2* receptor. *ADAM17* expression is increased in diabetes via *TNFα*. Furthermore, Furin, a member of the proportion convertase family, facilitates cellular entry and trafficking of ACE2 receptor [16]. We found that these genes were up-regulated in diabetic human heart (Fig. 4) and also in diabetic mouse (Fig. 6) possibly explaining higher ACE2 expression in diabetic heart and serum. We found significant correlation between ACE2 expression and this genes (Fig. 4e, g). Expression of genes effecting ACE2 trafficking positively correlate with glycemic control (Fig. 4f, g).

### Relationship between *ACE2* and *ACE1*

**Fig. 5:**
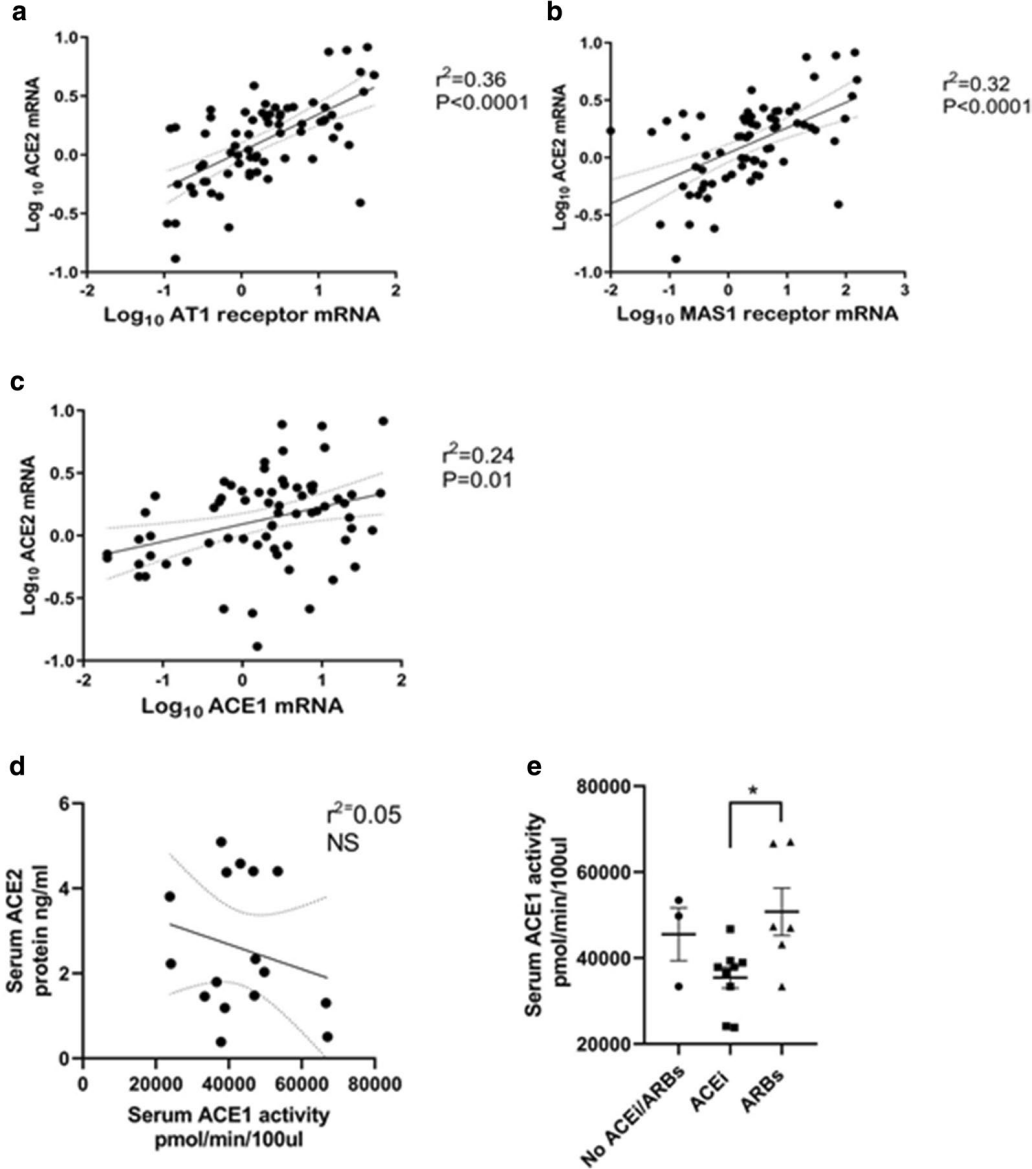
Correlation between ACE2 in from cardiac tissue biopsies and genes from RAS system. Results are presented as the Log10 of the normalized mRNA fold induction. **a** Direct correlation between ACE2 mRNA expression and MAS1 receptor mRNA. **b** Direct correlation between ACE2 and AT1 receptor mRNA in cardiac biopsies (n = 72). **c** Direct correlation between ACE2 and ACE1 mRNA. **d** No correlation between ACE2 and ACE1 activity. **e** Effect of ACEi, ARBs or no RAS blockers prior to the CABG operation on ACE1 activity in the serum (n = 19)

ACE2 is a potent counter-regulator against ACE1 activity and plays a protective role in the heart. The level of renin angiotensin system activation depends on *ACE1/ACE2* ratio [16, 29]. Increased ACE2 activate Ang-(1–7)/Mas receptor axis. We found a positive correlation between *ACE1* and *ACE2* mRNA in the heart biopsies. *ACE2* correlated with ANG II receptor expression and with downstream *MAS* receptor (Fig. 5).

Although ACE2 mRNA was significantly correlated to ACE1 at the mRNA level no correlation between ACE2 levels to ACE 1 activity levels was found in the serum (see Fig. 5d). ACE1 activity is decreased by treatment with ACEi (see Fig. 5e).

### Effect of angiotensin infusion on ACE2 expression in wild type and db/db mice

To elaborate on the observed results from human tissue we studied the effect of diabetes, obesity and angiotensin II on *ACE2* expression in the murine diabetic heart under experimental conditions.

**Fig. 6:**
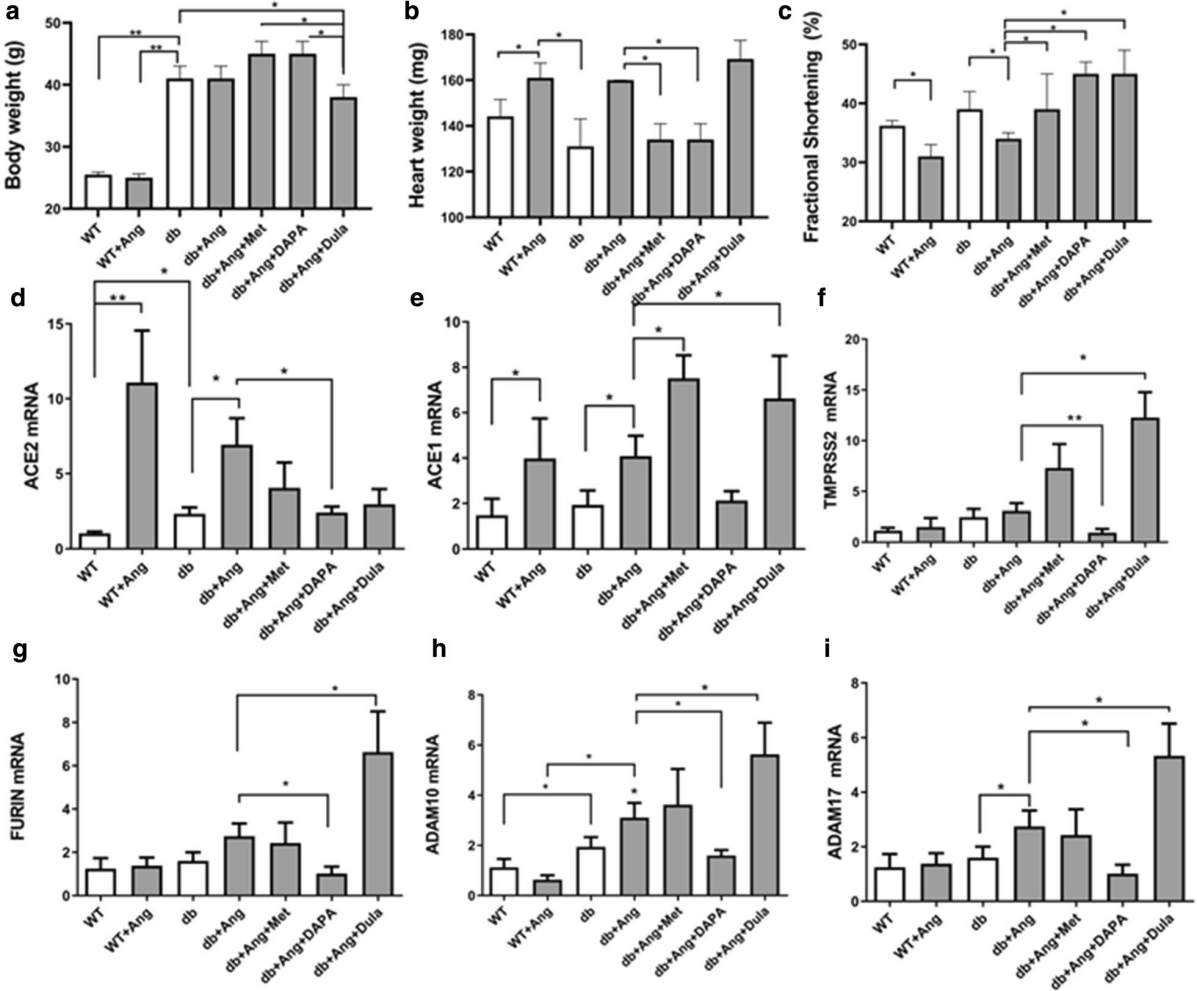
Body weight, heart weight, fractional shortening ACE2 and genes effecting ACE2 trafficking in mice treated (db/db) with antidiabetic drugs. Metformin (Met), Dapagliflozin (DAPA), Dulaglutide (Dula) were administered concomitantly with Ang. **a** Body weight, **b** heart weight and (**c**) fractional shortening are shown as mean ± SEM. mRNA expression levels of (**d**) ACE2, (**e**) ACE2 (**f**) *TMPRSS2* (**g**) *Furin* (**h**) *ADAM10*, (**I**) *ADAM17* in db/db (db) hearts following Ang infusion and anti-diabetic drugs. Values are shown as mean ± SEM P value evaluated with paired Student’s t-test, *p < 0.05**p < 0.001vs WT #P < 0.05 vs db/db mice

Diabetes in db/db mice was associated with severe obesity. AngII infusion resulted in cardiac hypertrophy in both WT and diabetic mice and a significant reduction in the fractional shortening. All three anti-diabetic drugs improved the fractional shortening (Fig. 6). Diabetic obese (db/db) mice have higher expression of ACE2 compare to WT mice.

Metformin (Met) and dapagliflozin (DAPA) did not reduce the body weight but were associated with a significant heart weight reduction. To the contrary, Dulaglutide (DULA) decreased the animal weight but not the heart weight (Fig. 6). Met, Dapa and DULA resulted in a decrease in ACE2 expression in ATII-stressed diabetic mice (Fig. 6).

## Discussion

Changes in *ACE2* expression has been suggested as the cause of variable sensitivity to SARS CoV-2 infection, viral infection mechanism and as a target for development of new anti-viral treatment strategies [30] Our study aimed to investigate *ACE2* mRNA and protein expression in human cardiac tissue and their correlation with COVID 19 infection risk factors: diabetes, drug therapy and cardiac function.

We like others have found that *ACE2* gene expression was increased in diabetic patients’ hearts compared to non-diabetic hearts [31]. It positively correlated with glycosylated hemoglobin (HbA1c), body mass index (BMI), and negatively correlated with the left ventricular ejection fraction and fibrosis. *ACE2* was not affected by age, the presence of LVH and treatment with ACEi or ARBs prior to the operation. We are the first to report that no correlation exist between plasma free *ACE2* and cardiac tissue *ACE2* expression. Transmembrane serine protease 2 (*TMPRSS2*)*, ADAM* metallopeptidase domain 10 and 17 (*ADAM10, ADAM17*) that facilitate viral *ACE2* complex entry and degradation were upregulated in diabetic hearts. As presented in Table 1, 95% of our patient are male in the non diabetic patients and 86% of the diabetic group were male, therefore we are unable to address the issue of sex difference. Furthermore the age interval is narrow therefore, we did not find a correlation age and ACE2. Analyzing the effect of glycemic control on ACE2 expression (Fig. 4) strongly suggest a positive correlation between HbA1C and ACE2 suggesting that variability in the human ACE2 expression is probably secondary to glycemic control.

According to several studies, there is an association between type 2 diabetes, obesity, heart failure and severity COVID-19 infection [44]. There are evidence of increased incidence and severity of COVID-19 in patients with diabetes with uncontrolled glycemic index [32]. The cause of uncontrolled inflammatory and immune response seen in diabetic and obese patients are still remains largely unknown and may possibly related to diabetic inflammatory or hormonal stat, higher SARS-CoV2 viral load, or associated with high expression of the ACE2 receptor [13].

An activation of cardiac *ACE2* occurs, in humans and rats following myocardial infarction [33]. We found that *ACE2* mRNA was highly expressed in the cardiac biopsies, and that uncontrolled diabetes with high HbA1c was an important factor that determined *ACE2* expression in the heart. Furthermore, IHC and Elisa *ACE2* protein assay analysis showed a higher protein expression in diabetic hearts and a correlation between *ACE2* and HbA1c.

The mechanism of up-regulation of *ACE2* in diabetes and obesity has not been fully studied. Possible mechanisms include: diabetic environment, hyperglycemia, inflammation: and inflammatory cytokine [34, 35], androgen stimulation [36], or the use of different medications effecting RAS [37].

From the start of the COVID-19 pandemic a concern was raised regarding the safety of RAS blockers use in high risk patients. ACEi and ARBs are known to increase the levels of *ACE2* by inhibiting the conversion of Ang I to Ang II [37]. It has been proposed that diabetic patients receiving ACEi or ARBs may have higher susceptibility to COVID-19 [3] and that diabetic patient with or without heart failure should be switched to other drugs during the pandemic. We found that *ACE2* mRNA and protein in cardiac tissue and also in serum was not differentially expressed between patients that had been taking ACEi or ARBs prior to the operation or not, albeit in a small group of patients, suggesting that ACEi or ARB scan be safely used during COVID19 infection. Our findings in cardiac tissue are in agreement with the recent publications showing that the use of renin–angiotensin–aldosterone inhibition appears protective for patients that continued taking these drugs during COVID19 infection compared with patients that discontinued RAS blockade [38–41]. The Council on Hypertension of the European Society of Cardiology recommended that patients should continue treatment with their usual anti-hypertensive therapy because there is no clinical or scientific evidence to suggest the discontinuation of ACEI or ARBs because of COVID-19 infection (https://www.escardio.org/Councils/Council-on-Hypertension).

Most publications on ACE2 expression in diabetes and obesity have been based on studying free *ACE2* in serum or plasma [27, 42–44]. We found no correlation between serum levels of free *ACE2* and cardiac tissue *ACE2* showing that it may be impossible to estimate tissue levels from serum levels.

Different anti-diabetic medications including insulin could affect *ACE2* availability in the respiratory tract and the heart promoting increased SARS-CoV2 uptake [30]. According to the literature all anti-diabetic drugs have been found to augment *ACE2* activity in the lung. Metformin, which is widely used to treat diabetes, increases *ACE2* expression by phosphorylation at Ser680 residue in HUVEC cells. AMPK-mediated phosphorylation of *ACE2*, induced by Metformin, improves ACE2 stability by hampering its ubiquitination and proteasomal degradation [45, 46]. Clinical experiments showed that the combined use of SGLT2i and ACEI/ARB significantly increased intra-renal *ACE2* expression. GLP1 elevated *ACE2* expression and activity in the rat heart [47, 48]. However, a recent large study in the UK showed that SGLT2i or DPP4 showed no influence on the susceptibility to COVID-19 infection [21]. Since we could not answer this question in humans due to concomitant therapies, we treated ATII-stressed diabetic mice with metformin, SGLT2 antagonist or GLP1 agonist. These mice were previously shown by us to develop cardiomyopathy associated with inflammation and fibrosis [22]. All three therapies resulted in reduced ACE2 expression concomitant with an improvement if systolic function.

Viral entry may be facilitated by hyperglycemia, which up-regulates ACE2 expression on cell surfaces and play a role in the progression to severe illness [49]. We found positive correlation between HbA1c and *ACE2. ACE2* activity was also increased in different models of diabetes and in the hearts of db/db nice as well as streptozocin induced diabetes [50]. We studied *ACE2* expression in db/db mice with angiotensin perfusion. The diabetic mice showed higher *ACE2* mRNA compared with WT mice that are lean and non-diabetic. Angiotensin II perfusion increased *ACE2* mRNA in both WT and db/db mice, but there is a possibility that the net effect is not up-regulation of *ACE2* protein or activity, since angiotensin II also mediates *ACE2* internalization and degradation [54]. The anti-diabetic medications Metformin, SGLT2i and GLP-1 agonists did not further augment *ACE2* mRNA (Fig. 6). Thus, our results in diabetic mice that antidiabetic drugs attenuated the upregulation of ACE2 support our human findings that glycemic control is associated with reduced ACE2 levels.

ACE2 is regulated by proteolytic enzyme [51]. Studies have shown that Ang-(1–7) by acting via Mas receptor exerts inhibitory effects on inflammation and anti-fibrosis effect [52]. We showed that mRNA of *TMPRSS2* and *ADAM10, ADAM17* and *Furin as well as MAS* were all up-regulated in the diabetic heart and may increase receptor internalization and shedding in diabetes (Figs. 4, 5). *ADAM-17* activity is up-regulated by binding of SARS-CoV2 to ACE2, facilitating viral entry [49]. In diabetes *ADAM-17* is activated [50]. Ang II-infusion has been shown to stimulate ACE2 mRNA as well as proteolytic shedding by *ADAM-17* and to net suppresses ACE2 activity [53]. The correlation data for *ADAM 10, TMPRSS2, ADAM17*, and *Furin*, relative to diabetes or no diabetes levels is bifurcate. For each of these there are subjects similar to no DM and a group of patients with much higher levels. The variability in the results probably depends on glycemic control and obesity. Our mice study showed that angiotensin increased the levels of *ACE*2 as well as the expression of genes effecting ACE2 trafficking. The anti-diabetic drugs tends to reduce ACE2 but not all the genes affecting *ACE2* trafficking. It is documented that patients with higher biochemical risk factors which are associated both with the serum *ACE2* level and a higher risk for mortality and cardiovascular disease, might contribute to better identification of risk for severe COVID-19 infection [31]. As shown in Table 1 all patients in this study were elective and do not suffer from the severe LV dysfunction. Furthermore, patient in this study were not infected with COVID.

ACE2 expression increases concomitantly with pathological states associated with activation of RAAS system. It has an anti-inflammatory and a protective function by down-regulating the RAS system. Up-regulation of ACE2 receptors may facilitate COVID-19 viral entry into cells, thereby increasing the susceptibility to infection. However, ACE2 down-regulation, after infection, is also thought to aggravate inflammation and cytokine storm.

## Conclusion

We studied the expression of ACE2 in a large cohort of human heart tissues with no SARS-cov2 infection. We demonstrated that ACE2 expression is increased in diabetes and obesity. No correlation was found with ACEi or ARBs that may be safely continued during COVID19 infection. ACE2 expression in mice was increased with ATII infusion and attenuated following anti-diabetic drugs treatment. Our findings may explain the susceptibility to COVID-19 infection in diabetes and obesity and suggest that diabetic control may modify the increased risk of infection in diabetic obese patients.

## Data Availability

All data and materials are available upon request.

## References

[1] Burrell LM, Johnston CI, Tikellis C, Cooper ME (2004). ACE2, a new regulator of the renin–angiotensin system. Trends Endocrinol Metab.

[2] Cheng H, Wang Y, Wang GQ (2020). Organ-protective effect of angiotensin-converting enzyme 2 and its effect on the prognosis of COVID-19. J Med Virol.

[3] Lew RA, Warner FJ, Hanchapola I, Yarski MA, Ramchand J, Burrell LM, Smith AI (2008). Angiotensin-converting enzyme 2 catalytic activity in human plasma is masked by an endogenous inhibitor. Exp Physiol.

[4] Basu R, Poglitsch M, Yogasundaram H, Thomas J, Rowe BH, Oudit GY (2017). Roles of angiotensin peptides and recombinant human ACE2 in heart failure. J Am Coll Cardiol.

[5] Epelman S, Tang WH, Chen SY, Van Lente F, Francis GS, Sen S (2008). Detection of soluble angiotensin-converting enzyme 2 in heart failure: insights into the endogenous counter-regulatory pathway of the renin–angiotensin–aldosterone system. J Am Coll Cardiol.

[6] Narula S, Yusuf S, Chong M, Ramasundarahettige C, Rangarajan S, Bangdiwala SI, van Eikels M, Leineweber K, Wu A, Pigeyre M (2020). Plasma ACE2 and risk of death or cardiometabolic diseases: a case-cohort analysis. Lancet.

[7] Soldo J, Heni M, Konigsrainer A, Haring HU, Birkenfeld AL, Peter A (2020). Increased hepatic ACE2 expression in NAFL and diabetes—a risk for COVID-19 patients?. Diabetes Care.

[8] Ceriello A, Standl E, Catrinoiu D, Itzhak B, Lalic NM, Rahelic D, Schnell O, Skrha J, Valensi P (2020). Issues for the management of people with diabetes and COVID-19 in ICU. Cardiovasc Diabetol.

[9] Liu H, Gai S, Wang X, Zeng J, Sun C, Zhao Y, Zheng Z (2020). Single-cell analysis of SARS-CoV-2 receptor ACE2 and spike protein priming expression of proteases in the human heart. Cardiovasc Res.

[10] Thum T (2020). SARS-CoV-2 receptor ACE2 expression in the human heart: cause of a post-pandemic wave of heart failure?. Eur Heart J.

[11] Inciardi RM, Adamo M, Lupi L, Cani DS, Di Pasquale M, Tomasoni D, Italia L, Zaccone G, Tedino C, Fabbricatore D (2020). Characteristics and outcomes of patients hospitalized for COVID-19 and cardiac disease in Northern Italy. Eur Heart J.

[12] Parohan M, Yaghoubi S, Seraji A (2020). Cardiac injury is associated with severe outcome and death in patients with coronavirus disease 2019 (COVID-19) infection: a systematic review and meta-analysis of observational studies. Eur Heart J Acute Cardiovasc Care.

[13] Cao Y, Li L, Feng Z, Wan S, Huang P, Sun X, Wen F, Huang X, Ning G, Wang W (2020). Comparative genetic analysis of the novel coronavirus (2019-nCoV/SARS-CoV-2) receptor ACE2 in different populations. Cell Discov.

[14] Sattar N, McInnes IB, McMurray JJV (2020). Obesity is a risk factor for severe COVID-19 infection: multiple potential mechanisms. Circulation.

[15] Zavascki AP, Falci DR (2020). Clinical characteristics of Covid-19 in China. N Engl J Med.

[16] Hoffmann M, Kleine-Weber H, Schroeder S, Kruger N, Herrler T, Erichsen S, Schiergens TS, Herrler G, Wu NH, Nitsche A (2020). SARS-CoV-2 cell entry depends on ACE2 and TMPRSS2 and is blocked by a clinically proven protease inhibitor. Cell.

[17] Domenighetti AA, Wang Q, Egger M, Richards SM, Pedrazzini T, Delbridge LM (2005). Angiotensin II-mediated phenotypic cardiomyocyte remodeling leads to age-dependent cardiac dysfunction and failure. Hypertension.

[18] Podgoreanu MV, White WD, Morris RW, Mathew JP, Stafford-Smith M, Welsby IJ, Grocott HP, Milano CA, Newman MF, Schwinn DA (2006). Inflammatory gene polymorphisms and risk of postoperative myocardial infarction after cardiac surgery. Circulation.

[19] Meng J, Xiao G, Zhang J, He X, Ou M, Bi J, Yang R, Di W, Wang Z, Li Z (2020). Renin–angiotensin system inhibitors improve the clinical outcomes of COVID-19 patients with hypertension. Emerg Microbes Infect.

[20] Yang G, Tan Z, Zhou L, Yang M, Peng L, Liu J, Cai J, Yang R, Han J, Huang Y (2020). Effects of angiotensin II receptor blockers and ACE (angiotensin-converting enzyme) inhibitors on virus infection, inflammatory status, and clinical outcomes in patients with COVID-19 and hypertension: a single-center retrospective study. Hypertension.

[21] Sainsbury C, Wang J, Gokhale K, Acosta-Mena D, Dhalla S, Byne N, Chandan JS, Anand A, Cooper J, Okoth K (2020). Sodium-glucose-co-transporter-2 inhibitors and susceptibility to COVID-19: a population-based retrospective cohort study. Diabetes Obes Metab.

[22] Waldman M, Cohen K, Yadin D, Nudelman V, Gorfil D, Laniado-Schwartzman M, Kornwoski R, Aravot D, Abraham NG, Arad M (2018). Regulation of diabetic cardiomyopathy by caloric restriction is mediated by intracellular signaling pathways involving 'SIRT1 and PGC-1alpha'. Cardiovasc Diabetol.

[23] Arow M, Waldman M, Yadin D, Nudelman V, Shainberg A, Abraham NG, Freimark D, Kornowski R, Aravot D, Hochhauser E (2020). Sodium-glucose cotransporter 2 inhibitor Dapagliflozin attenuates diabetic cardiomyopathy. Cardiovasc Diabetol.

[24] Eskens BJ, Zuurbier CJ, van Haare J, Vink H, van Teeffelen JW (2013). Effects of two weeks of metformin treatment on whole-body glycocalyx barrier properties in db/db mice. Cardiovasc Diabetol.

[25] Kimura T, Obata A, Shimoda M, Hirukawa H, Kanda-Kimura Y, Nogami Y, Kohara K, Nakanishi S, Mune T, Kaku K (2018). Durability of protective effect of dulaglutide on pancreatic beta-cells in diabetic mice: GLP-1 receptor expression is not reduced despite long-term dulaglutide exposure. Diabetes Metab.

[26] Skarstein Kolberg E (2020). ACE2, COVID19 and serum ACE as a possible biomarker to predict severity of disease. J Clin Virol.

[27] Ciaglia E, Vecchione C, Puca AA (2020). COVID-19 infection and circulating ACE2 levels: protective role in women and children. Front Pediatr.

[28] Emilsson V, Gudmundsson EF, Aspelund T, Jonsson BG, Gudjonsson A, Launer LJ, Jennings LL, Gudmundsdottir V, Gudnason V (2020). Antihypertensive medication uses and serum ACE2 levels: ACEIs/ARBs treatment does not raise serum levels of ACE2. medRxiv.

[29] Kittana N (2018). Angiotensin-converting enzyme 2-Angiotensin 1–7/1-9 system: novel promising targets for heart failure treatment. Fundam Clin Pharmacol.

[30] Rahimi L, Malek M, Ismail-Beigi F, Khamseh ME (2020). Challenging issues in the management of cardiovascular risk factors in diabetes during the COVID-19 pandemic: a review of current literature. Adv Ther.

[31] Wallentin L, Lindback J, Eriksson N, Hijazi Z, Eikelboom JW, Ezekowitz MD, Granger CB, Lopes RD, Yusuf S, Oldgren J (2020). Angiotensin-converting enzyme 2 (ACE2) levels in relation to risk factors for COVID-19 in two large cohorts of patients with atrial fibrillation. Eur Heart J.

[32] Onder G, Rezza G, Brusaferro S (2020). Case-fatality rate and characteristics of patients dying in relation to COVID-19 in Italy. JAMA.

[33] Burrell LM, Risvanis J, Kubota E, Dean RG, MacDonald PS, Lu S, Tikellis C, Grant SL, Lew RA, Smith AI (2005). Myocardial infarction increases ACE2 expression in rat and humans. Eur Heart J.

[34] Ziegler CGK, Allon SJ, Nyquist SK, Mbano IM, Miao VN, Tzouanas CN, Cao Y, Yousif AS, Bals J, Hauser BM (2020). SARS-CoV-2 receptor ACE2 is an interferon-stimulated gene in human airway epithelial cells and is detected in specific cell subsets across tissues. Cell.

[35] Onabajo OO, Banday AR, Yan W, Obajemu A, Stanifer ML, Santer DM, Florez-Vargas O, Piontkivska H, Vargas J, Kee C et al. Interferons and viruses induce a novel primate-specific isoform dACE2 and not the SARS-CoV-2 receptor ACE2. bioRxiv. 2020. 10.1038/s41588-020-00731-9 PMC9377523 33077916

[36] Mjaess G, Karam A, Aoun F, Albisinni S, Roumeguere T (2020). COVID-19 and the male susceptibility: the role of ACE2, TMPRSS2 and the androgen receptor. Prog Urol.

[37] South AM, Brady TM, Flynn JT (2020). ACE2 (angiotensin-converting enzyme 2), COVID-19, and ACE inhibitor and Ang II (angiotensin II) receptor blocker use during the pandemic: the pediatric perspective. Hypertension.

[38] Caldeira D, Alves M, Gouveia EMR, Silverio Antonio P, Cunha N, Nunes-Ferreira A, Prada L, Costa J, Pinto FJ (2020). Angiotensin-converting enzyme inhibitors and angiotensin-receptor blockers and the risk of COVID-19 infection or severe disease: systematic review and meta-analysis. Int J Cardiol Heart Vasc.

[39] Mancia G, Rea F, Ludergnani M, Apolone G, Corrao G (2020). Renin–angiotensin–aldosterone system blockers and the risk of Covid-19. N Engl J Med.

[40] Hippisley-Cox J, Tan PS, Coupland C (2020). Risk of severe COVID-19 disease with ACE inhibitors and angiotensin receptor blockers: cohort study including 8.3 million people. Heart.

[41] Mehra MR, Desai SS, Kuy S, Henry TD, Patel AN (2020). Cardiovascular disease, drug therapy, and mortality in Covid-19. N Engl J Med.

[42] Anguiano L, Riera M, Pascual J, Soler MJ (2017). Circulating ACE2 in cardiovascular and kidney diseases. Curr Med Chem.

[43] Uri K, Fagyas M, Kertesz A, Borbely A, Jenei C, Bene O, Csanadi Z, Paulus WJ, Edes I, Papp Z (2016). Circulating ACE2 activity correlates with cardiovascular disease development. J Renin Angiotensin Aldosterone Syst.

[44] Soro-Paavonen A, Gordin D, Forsblom C, Rosengard-Barlund M, Waden J, Thorn L, Sandholm N, Thomas MC, Groop PH, FinnDiane Study G (2012). Circulating ACE2 activity is increased in patients with type 1 diabetes and vascular complications. J Hypertens.

[45] Zhang J, Dong J, Martin M, He M, Gongol B, Marin TL, Chen L, Shi X, Yin Y, Shang F (2018). AMP-activated protein kinase phosphorylation of angiotensin-converting enzyme 2 in endothelium mitigates pulmonary hypertension. Am J Respir Crit Care Med.

[46] Malhotra A, Hepokoski M, McCowen KC, John YJS (2020). ACE2, metformin, and COVID-19. iScience.

[47] Zhang LH, Pang XF, Bai F, Wang NP, Shah AI, McKallip RJ, Li XW, Wang X, Zhao ZQ (2015). Preservation of glucagon-like peptide-1 level attenuates angiotensin II-induced tissue fibrosis by altering AT1/AT 2 receptor expression and angiotensin-converting enzyme 2 activity in rat heart. Cardiovasc Drugs Ther.

[48] Romani-Perez M, Outeirino-Iglesias V, Moya CM, Santisteban P, Gonzalez-Matias LC, Vigo E, Mallo F (2015). Activation of the GLP-1 receptor by liraglutide increases ACE2 expression, reversing right ventricle hypertrophy, and improving the production of SP-A and SP-B in the lungs of type 1 diabetes rats. Endocrinology.

[49] Sardu C, D'Onofrio N, Balestrieri ML, Barbieri M, Rizzo MR, Messina V, Maggi P, Coppola N, Paolisso G, Marfella R (2020). Outcomes in patients with hyperglycemia affected by COVID-19: can we do more on glycemic control?. Diabetes Care.

[50] Wysocki J, Ye M, Soler MJ, Gurley SB, Xiao HD, Bernstein KE, Coffman TM, Chen S, Batlle D (2006). ACE and ACE2 activity in diabetic mice. Diabetes.

[51] Heurich A, Hofmann-Winkler H, Gierer S, Liepold T, Jahn O, Pohlmann S (2014). TMPRSS2 and ADAM17 cleave ACE2 differentially and only proteolysis by TMPRSS2 augments entry driven by the severe acute respiratory syndrome coronavirus spike protein. J Virol.

[52] Simoes e Silva AC, Silveira KD, Ferreira AJ, Teixeira MM (2013). ACE2, angiotensin-(1–7) and Mas receptor axis in inflammation and fibrosis. Br J Pharmacol.

[53] Gross S, Jahn C, Cushman S, Bar C, Thum T (2020). SARS-CoV-2 receptor ACE2-dependent implications on the cardiovascular system: from basic science to clinical implications. J Mol Cell Cardiol.

